# Supplementary material for: The neuronal ceroid lipofuscinosis protein Cln7 functions in the postsynaptic cell to regulate synapse development
Source: Sci Rep. 2019 Oct 30;9:15592. doi: 10.1038/s41598-019-51588-w (PMC6821864; doi:10.1038/s41598-019-51588-w)
Supplement: Supplementary file 1 — Supplementary Information [file 41598_2019_51588_MOESM1_ESM.pdf]

**The neuronal ceroid lipofuscinosis protein Cln7 functions in the postsynaptic cell to regulate synapse development.**

Kyle J. Connolly, Megan B. O’Hare, Alamin Mohammed, Katelyn M. Aitchison, Niki C. Anthoney, Matthew J. Taylor, Bryan A. Stewart, Richard I. Tuxworth and Guy Tear

## Supplementary figures

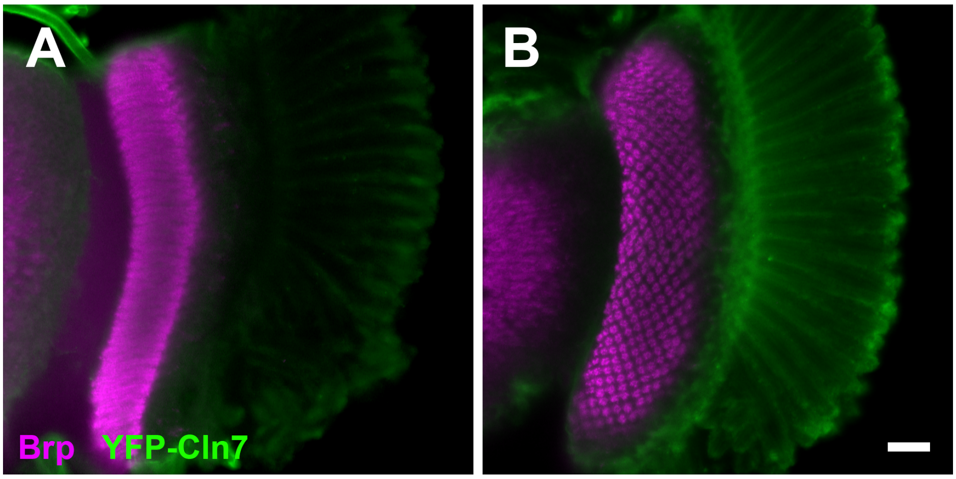


**Figure S1. *Cln7* is expressed in neurons of the adult visual system.**
Confocal images of the adult retina of *w*[1118] control (A) and YFP-Cln7 knock-in (B) flies. Active zones in synapses were stained with anti-Brp (magenta) and YFP-Cln7 with anti-GFP (green). Scale bar = 20 μm

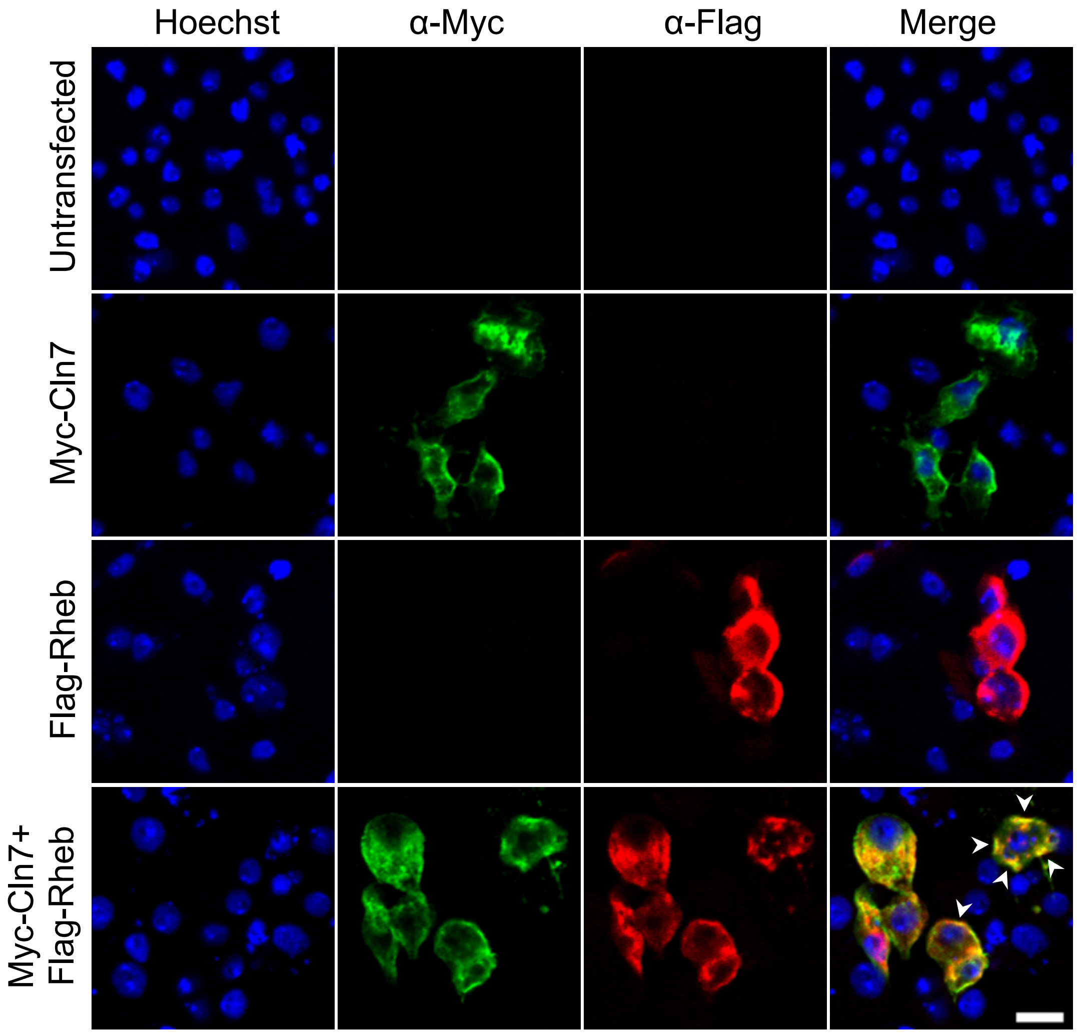


**Figure S2. Cln7 co-localises with Rheb.**
Protein localisation was analysed in single- and double-transfected cells by immunofluorescence. Arrowheads show areas of co-localisation. Scale bar = 10 μm.
